# A fully sustainable, self-poled, bio-waste based piezoelectric nanogenerator: electricity generation from pomelo fruit membrane

**DOI:** 10.1038/s41598-020-68751-3

**Published:** 2020-07-21

**Authors:** Satyaranjan Bairagi, Saikat Ghosh, S. Wazed Ali

**Affiliations:** Department of Textile and Fibre Engineering, Indian Institute of Technology Delhi, New Delhi, 110016 India

**Keywords:** Materials science, Actuators, Electronic devices, Sensors and biosensors

## Abstract

A self-powered system is very much essential aspect in the recent trend to improve the working efficiency of the portable and wearable devices. Here, we have reported a fully sustainable, self-poled, bio-compatible, and bio-waste based piezoelectric energy harvester which has been made of Pomelo Fruit Membrane (PFM). PFM based piezoelectric generator (PFMBPEG) could generate ~ 6.4 V output voltage and ~ 7.44 μA output current directly, only by finger tapping on the device and registers a power density of ~ 12 μW cm^−2^ whereas, the same piezoelectric generator can generate ~ 15 V output voltage, 130 μA output current, and power density of ~ 487.5 μW cm^−2^ by using a full wave rectifier. The sensitivity and energy harvesting competence of the generator have also been assessed by attaching this nanogenerator into various parts of human body (as energy sources) such as wrist, elbow, finger, throat, jaws, leg and putting the device into ultrasonic bath and in every case, it could successfully generate voltage. Therefore, this bio-waste based energy harvester can be used as a power source for the different potable and wearable electronic goods where a small amount of energy is required, specifically in the biomedical applications (i.e., health monitoring, power source for the implantable devices and so on). Finally, mechanical stability the developed piezoelectric generator has been evaluated by cyclic bending test and it has been observed that there is no significant deformation of the PFM film even after 100 cycles.

## Introduction

A self-powered system is a promising demand in our daily life, to avoid disadvantages of the battery (battery has a limited life time, charging problem). Also, self-powered system would increase the work ability in real time applications^1–3^. Fossil fuel is a large source of energy for the different electronic systems, but it has enlarged the environmental pollution. Due to this environmental issue, nowadays research on the clean energy has drawn a great interest. Among the different clean energies, mechanical energy would be the prime candidate for the self-powered systems, because of its sustainability and tremendous availability in the surroundings^4–7^. Mechanical energy can exist in different forms such as vibration, wind, sound wave, flow of water and energy from human body movement, etc. which are mainly waste energies. The best technology for the self-powered system till date is piezoelectric material-based technology than others (triboelectric, electromagnetic, electrostatic, etc.)^8^.

The selection of piezoelectric materials is one of the important tasks to maintain all the desirable properties such as flexibility, eco-friendliness and easy availability. Research has already been carried out by using different inorganic and organic based piezoelectric materials for instance, lead zirconate titanate (PZT)^9^, barium titanate (BaTiO_3_)^10,11^, zinc oxide (ZnO)^12^, zinc stannate (ZnSnO_3_)^13^, potassium sodium niobite (KNN)^14–16^, sodium niobite (NaNbO_3_), poly (vinylidene difluoride) (PVDF)^17^ and its co-polymers. Moreover, different composite based piezoelectric materials have also been explored in the recent trend. However, some of the above-mentioned piezoelectric materials, have some drawbacks because of their toxic nature, non-ecofriendliness, non-biocompatibility, non-biodegradability, rigidity, and lower piezoelectric properties. In addition, piezoelectric materials development involves a long and hazardous chemical step for their synthesis and fabrication which are their other limitations. Such drawbacks of these materials retired them from being used in the biomedical applications. Furthermore, for some techniques of the fabrication of piezoelectric materials like solution cast, melt-extrusion, an electrical poling process is essential for getting better piezoelectric effect. The use of poling arrangement is also a big challenging task for the materials manufacturers.

To avoid the above limitations of the different available piezoelectric materials, discovering of bio-based piezoelectric materials is a main moto to the recent research community. A surge of research has already been demonstrated by different research groups. It has been found that waste fish scale and fish bladder have been used as a piezoelectric material in a research^18,19^. They have discovered the piezoelectric effect in this bio-waste material, and they found 10 V output voltage and 51 nA current from this waste-based material. In another study, authors have found out the piezoelectric properties in the waste onion skin^20^. This onion skin based piezoelectric generator could generate 18 V output voltage, 166 nA output current and 1.7 μW cm^−2^ power density simply by finger tapping on the generator. These findings lead the researcher to get motivation for further exploring on the bio-waste based piezoelectric generator.

In this study, we have discovered Pomelo Fruit Membrane (PFM) as a fully sustainable, self-poled and bio-waste based piezoelectric material. Pomelo fruit membrane is composed of cellulose 1, hemicellulose and pectin-based polysaccharides. Generally, cellulose is a bio-polymer consist of β-1,4-glycosidic bonds which are present in every repeating unit of d-glucose^21^. Hemicellulose and pectin are basically amorphous component in the PFM. But they contain carboxylic, hydroxyl and carbonyl groups. Pectin is a heterogeneous polysaccharide comprising of a linear homoglacturonan namely α-1,4-galacturonic acids chain, which is interconnected with the branch of rhamnogalacturonan chains^21,22^. The complex network among these three components (cellulose, hemicellulose and pectin) in the pomelo fruit membrane (PFM) is responsible for the piezoelectric properties of this PFM. –H atoms of one component form strong hydrogen bonds with the –O atoms of the other component or them-self. These hydrogen bonds act as dipoles in the PFM.

As mentioned above, some of the researches have been done by using bio-based piezoelectric materials. But this is the first attempt to discover piezoelectric properties in sustainable, self-poled and bio-waste-based pomelo fruit membrane (PFM). The generator has been developed directly by using the pomelo fruit membrane collected from the market without further treatment. In addition, it has been found that this waste membrane based piezoelectric generator (PFMBPEG) directly can generate ~ 6.4 V output voltage, ~ 7.44 μA current and ~ 12 μW cm^−2^ power density while, by using a full wave rectifier, this generator can generate ~ 15 V output voltage, ~ 130 μA output current, and ~ 487.5 μW cm^−2^ power density. Moreover, for testing the practical applicability and sensitivity of the developed PFMBPEG, different energy sources such as elbow, wrist, fingers, leg, throat, jaws motion and ultrasonication wave have been used in this present study and it has been observed that this generator is very sensitive to all these movements even to a very small-scale vibration.

## Experimental section

### Collection of pomelo fruit membrane

The pomelo fruit (*Citrus maxima*) was purchased from the Green Park market, New Delhi, India. The fruit was washed several time using DI water and cut into pieces using knife. The membrane was collected from the fruit and washed several time using DI water followed by drying at room temperature for 1 day. This membrane is basically a waste part of the pomelo fruit.

### Fabrication of piezoelectric generator

**Figure 1:**
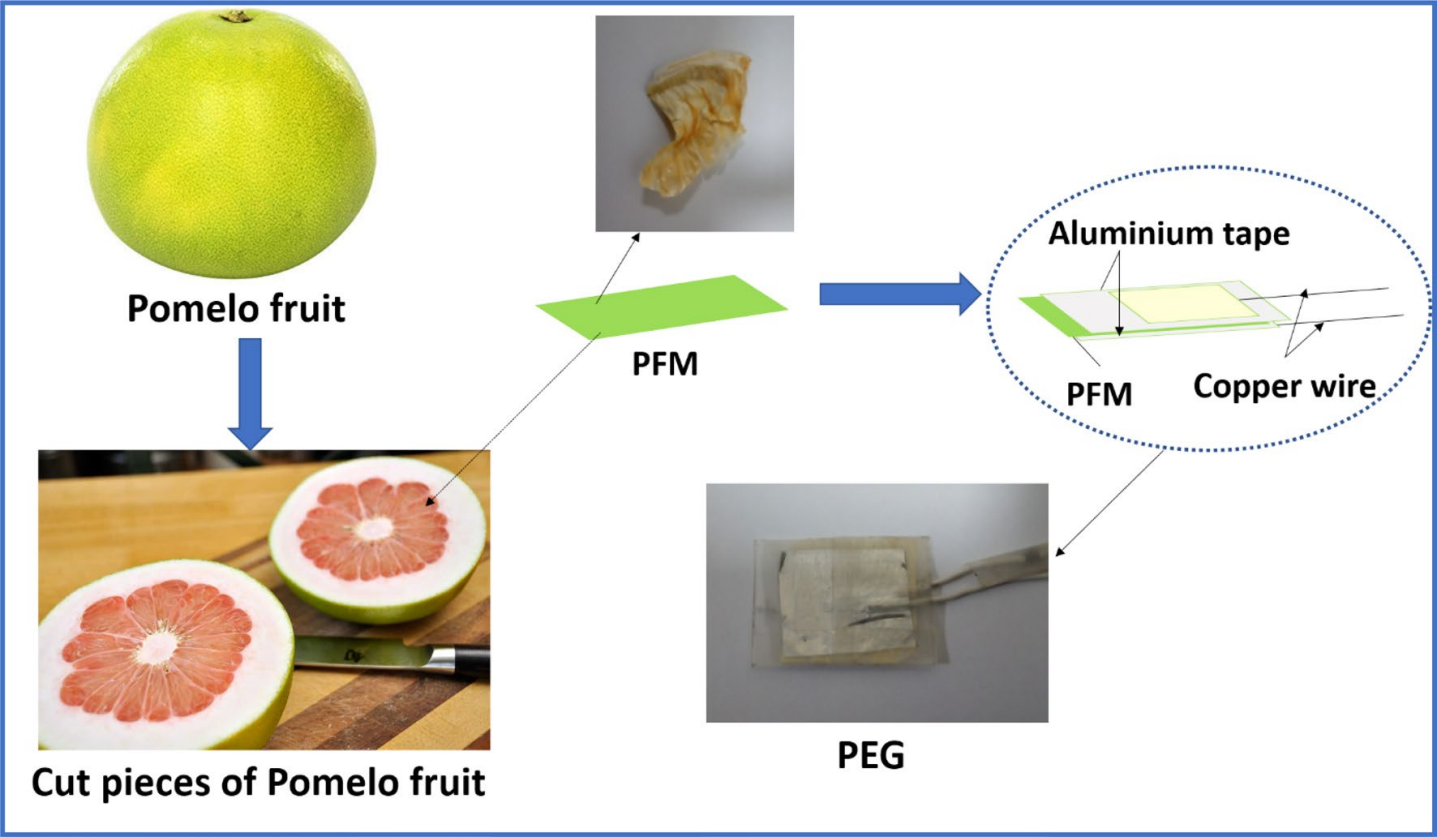
Schematic line diagram of fabrication of piezoelectric generator (PEG) based on pomelo fruit membrane (PFM).

The dried membrane of the pomelo fruit was cut into the dimensions of 2 × 2 × 0.045 cm^3^ for making a piezoelectric generator. The dried membrane was used to fabricate piezoelectric generator directly without further any treatments. Then conductive aluminium tape was pasted on both sides of the membrane which was acting as an electrode material. Finally, copper wires were connected to the membrane and encapsulated the whole device using polypropylene (PP) tape to protect the device from environmental issues. The schematic line diagram of the generator fabrication is shown in Fig. 1.

### Different characterizations

#### Fourier transform infrared (FTIR) spectroscopy

The different functional groups such as hydroxyl, carbonyl in the pomelo fruit membrane were identified by IR spectrum (FTIR, NICOLET 5700) with Attenuated Total Reflection (ATR) mode. The membrane was scanned in the wavenumber range of 400–4000 cm^−1^ with the 64 number of scans and a resolution of 4 cm^−1^.

#### X-ray diffraction (XRD)

The crystal structure and crystallinity of the membrane were calculated from the XRD (RIGAKU ULTIMA-IV, Japan) patterns. The membrane was scanned in the 2θ range of 10^ο^–60^ο^ with the scan speed of 4 cm^−1^ using CuKα X-ray radiation with 0.02^ο^ scan angle.

#### Scanning electron microscope (SEM)

The surface morphology of the pomelo fruit membrane was evaluated by SEM (SEM, ZEISS EVO 50) working with an accelerating voltage of 20 kV. To avoid the charging effect during sample scanning, membrane was gold (Au) coated by using the sputter coating unit.

#### Inductance capacitance resistance (LCR) meter

The dielectric properties (dielectric constant and loss) of the pomelo fruit membrane were taken by digital LCR meter (LCR, GWINSTEK, 8110G). The experiment was carried out by connecting two ends of the copper wires with the crocodile clips with an accelerating voltage of 10 mV.

#### Polarization-electric field (P-E) hysteresis

The ferroelectric behaviours (remnant polarization, coercive electrified, maximum polarization) of the pomelo fruit membrane were measured by P-E hysteresis (P-E, P-LC100V, RADIANT TECHNOLOGY PRECISION) loop analysis with a working frequency of 50 Hz.

#### Piezoelectric properties analysis

The output voltage and current were measured by using digital oscilloscope (DIGITAL OSCILLOSCOPE, ROHDE & SCHWARZ, RTB2002, India) by simple hand tapping on the device.

#### Evaluation of the mechanical stability of the piezoelectric device

To evaluate the mechanical and electrical stability of the developed PFM based generator a cyclic tensile test has been carried out using an Instron tensile tester (INSTRON 3365, UNIVERSAL TENSILE TESTING MACHINE). The testing was done with the following parameters: gauge length-of 40 mm, constant load of 400 gf and a test speed of 1 mm min^−1^ where the number of cycles was 100.

### Informed consent

The authors have attested to informed consent for publication of identifying information/images in an online open-access publication.

## Results and discussion

### Structural and morphological analysis of the PFM

**Figure 2:**
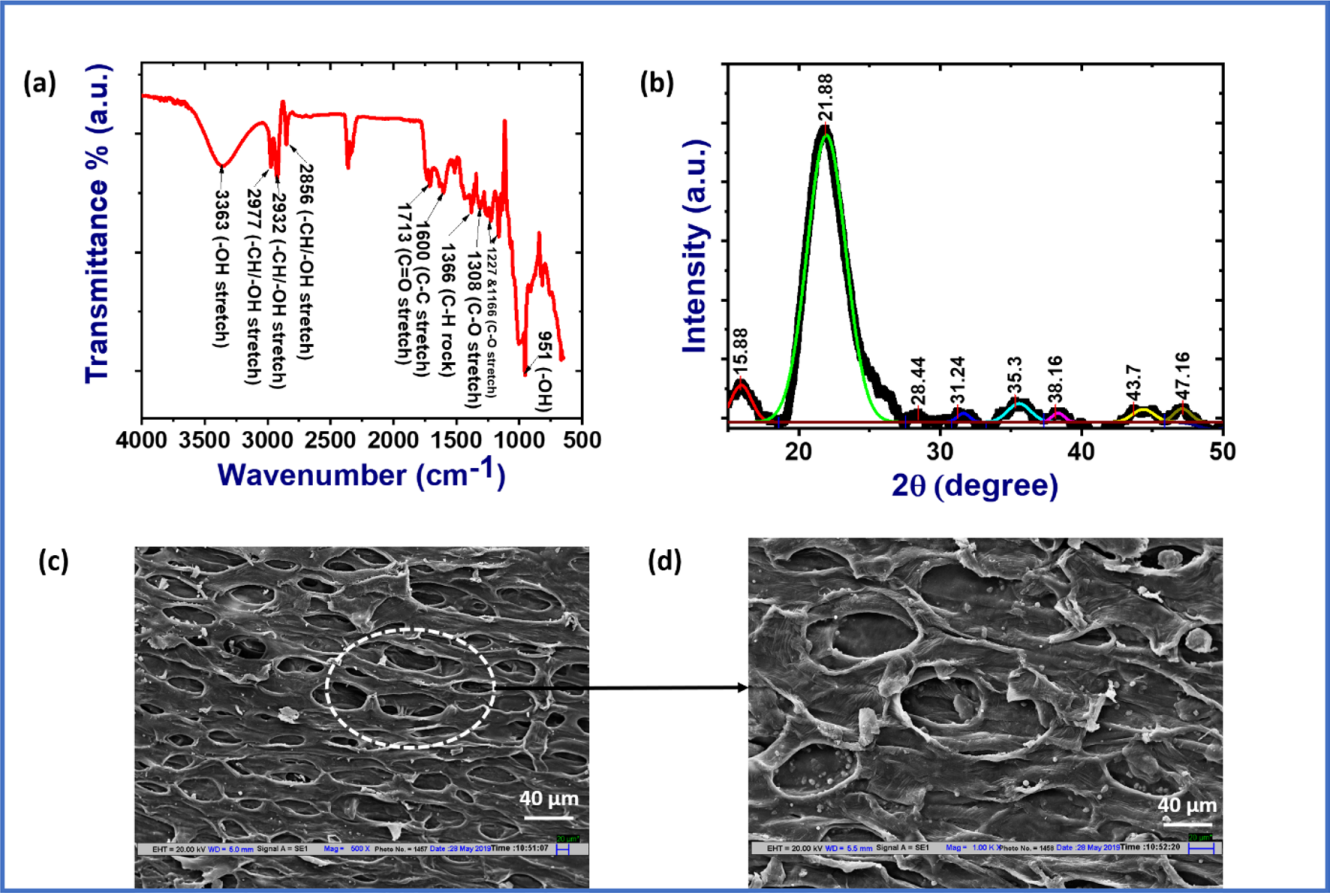
(**a**) IR spectrum of the pomelo membrane, (**b**) XRD patterns of the pomelo membrane, (**c**) surface morphology of the pomelo membrane, and (**d**) zoom view of the highlighted part of the (**c**).

The structural analysis of pomelo fruit membrane (PFM) has been carried out by IR spectrum as depicted in Fig. 2a. A sharp vibration peak appeared at the wavenumber of 951 cm^−1^ due to the bending of O–H functional groups present in the carboxylic acids of pectin component. Again, several weak absorption vibration peaks appeared at 1,166 cm^−1^, 1,227 cm^−1^, 1,308 cm^−1^ which are confirming the presence of carboxylic acids, alcohols and ether groups resulting from C–O stretching. The presence of alkane has been confirmed by the appearance of peak at 1,366 cm^−1^ as C–H rocking vibration occurs. Interestingly, C–C stretching happens at 1,600 cm^−1^ which indicates the ring structure. The presence of carbonyl groups have been confirmed by the peak at 1,710 cm^−1^. The presence of C–H stretching/bending vibration or O–H stretching vibration has been confirmed by the existence of absorption peaks at 2,856 cm^−1^, 2,932 cm^−1^ and 2,977 cm^−1^. A strong broad absorption peak appeared in the region of 3,350 to 3,360 cm^−1^, due to O–H stretching vibration, which strongly supports the existence of plenty of hydroxyl groups in pomelo fruit membrane^23–29^. Thus, pomelo fruit membrane (PFM) contains alcohols or hydroxyl groups, carbonyl groups, carboxylic acids, ethers and carbon atom which in turn confirm the structure of cellulose, hemicellulose and pectin in the membrane. Therefore, the presence of these functional groups in PFM helps to form strong hydrogen bonds among themselves, which results proper orientation of dipoles. Due to this dipole orientation a self-poled bio-based piezoelectric generator can be developed^30^.

For further investigation of structural frame of the PFM, XRD pattern has been analysed (Fig. 2b). It has been found that there are four diffraction peaks at 2θ values of 15.5^ο^, 17.12^ο^, 21.83^ο^, and 34.34^ο^ corresponding to the (− 1 1 0), (1 1 0), (2 0 0), and (0 0 4) crystallographic planes. These crystal planes are confirming the presence of cellulose I, hemicellulose and pectin components in the pomelo fruit membrane (PFM)^31–35^. The total crystallinity of the membrane is 47% as calculated from the XRD pattern using the below formula:

1 $$X_{{C_{t} }} = \frac{{\sum A_{cr} }}{{\sum A_{cr} + \sum A_{amr} }} \times 100\%$$

where, ${A}_{cr}$ and ${A}_{amr}$ are the crystalline and amorphous area of the membrane.

In general, the presence of cellulose components in the pomelo fruit membrane (PFM) have been responsible for piezoelectric property of the membrane. The –H atoms of one chain form hydrogen bonds with the –O atoms of the same chain or within the side chain. This hydrogen bonds provide net dipole moment during the application of pressure on the membrane and hence, piezoelectric effect has been observed from this pomelo fruit membrane (PFM). The complex network among the cellulose, hemicellulose and pectin component in the PFM is given in the supporting information (Fig. S1).

The surface morphology of the pomelo fruit membrane (PFM) has been observed by scanning electron microscope (SEM) working with an accelerating voltage of 20 kV. The surface morphology of the PFM is shown in Fig. 2c,d. Throughout the membrane surface small holes are present which connect each other by a small wall formed by cellulose microfibril, hemicellulose and pectin. From the Fig. 2d, a coating like structure throughout the surface along with small particles can also be observed. This compact structure may be due to the higher number of hydrogen bondings among the cellulose, hemicellulose and pectin components. However, hemicellulose and pectin are amorphous materials. In addition, all the small holes and their walls maintain a particular orientation in the overall membrane. This orientation may be another proof of dipoles alignment in PFM.

### Ferroelectric and dielectric properties analysis of the PFM

**Figure 3:**
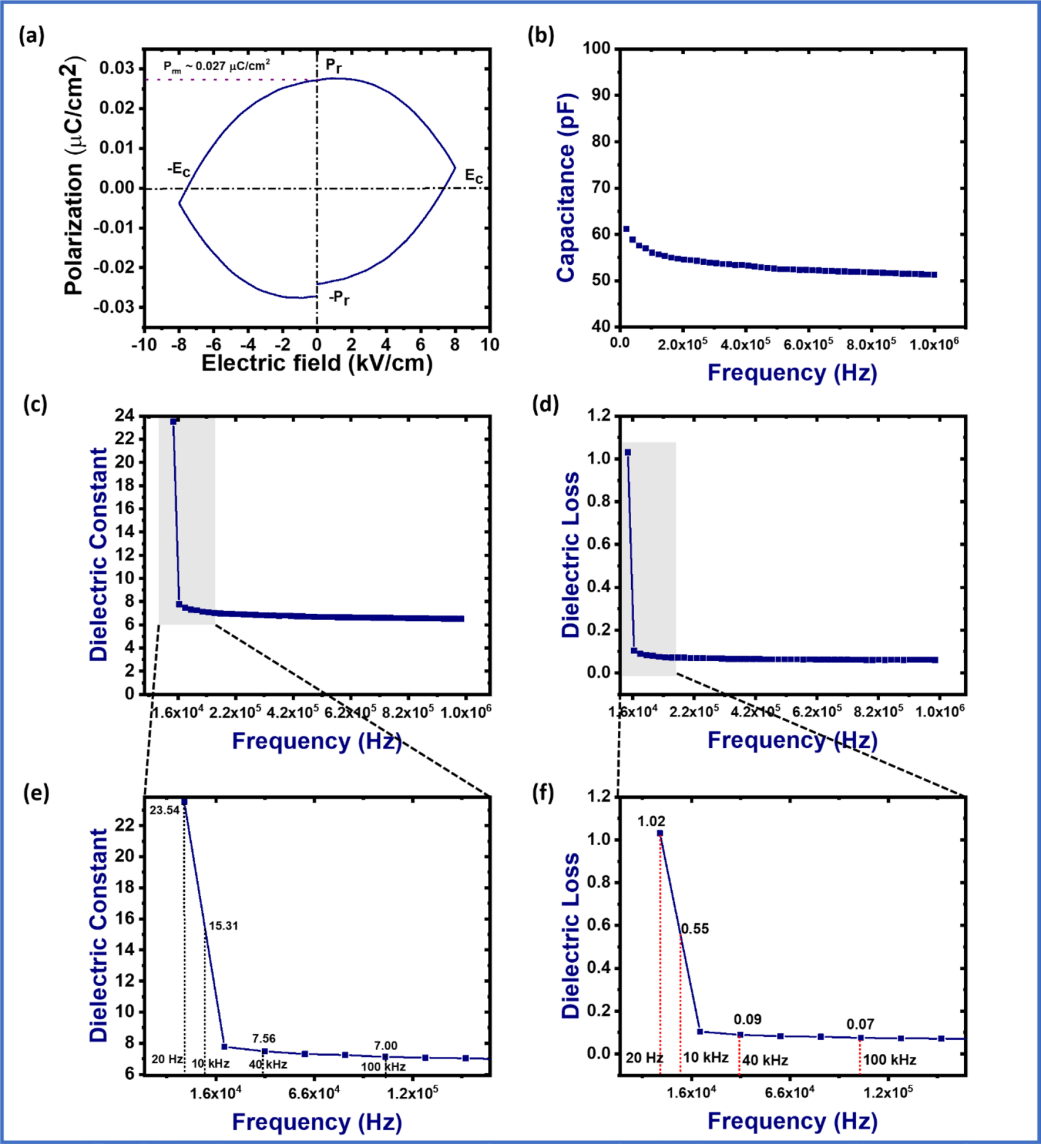
(**a**) P-E hysteresis loop, (**b**) frequency vs capacitance curve, (**c**) frequency vs dielectric constant curve, (**d**) frequency vs dielectric loss curve, (**e**) zoomed view of the selected part of (**c**), and (**f**) zoomed view of the selected part of (**d**).

The self-polarization behaviour of the pomelo fruit membrane (PFM) has been evaluated by using P-E hysteresis loop analysis which is shown in Fig. 3a. The remnant polarization observed was 0.027 μC cm^−2^ which is due to the intermolecular hydrogen bonding between the cellulose-cellulose, cellulose-hemicellulose, cellulose-pectin. In addition, polarization in the PFM may be due to the strong electrochemical interaction among the different components, by Maxwell stress^36^. Since, PFM is a natural material, the exact mechanism of piezoelectric and ferroelectric characteristics of membrane are not very clear. From Fig. 3a it can also be observed that the polarization value gets decreased with increasing the external electric field due to the disturbance in orientation of the dipoles inside the PFM film at higher electric field.

Furthermore, electrical characteristics of PFM, dielectric properties (dielectric constant and dielectric loss) have also been measured using digital LCR meter. For calculation of the dielectric constant value of PFM, first the capacitance value has been measured with respect to different frequencies as shown in Fig. 3b. Then the dielectric constant value has been calculated by using the following formula^37^:

2 $$\varepsilon = \frac{C \times d}{{\varepsilon_{0 \times A} }}$$

where, C, d are the capacitance and thickness of the PFM and ${\varepsilon }_{0}$, A are the relative permittivity at the void space (8.854 × 10^–12^ pF m^−1^) and area of the membrane, respectively.

Figure 3c–f show the dielectric constant and loss values of the pomelo fruit membrane having the frequency range of 20 Hz–1 MHz. The dielectric constant and loss value have been calculated as ~ 23.54 and 1.02, respectively. It’s very interesting to mention that the dielectric value of the PFM is higher than most usable piezoelectric polymer i.e. PVDF (8–10)^38^. It can be seen from the Fig. 3c,e that the dielectric constant value has been decreased with increasing in the frequency, which reveals the ferroelectric characteristics of the PFM^39^. Moreover, this higher dielectric constant and lower dielectric loss value implies the superior insulating nature of PFM. The dielectric constant and loss value have not been affected by the change of frequency at the higher range of frequency (40 kHz–1 MHz). This is due to the strong interaction between the cellulose and hemicellulose, cellulose–cellulose and cellulose–pectin or by other components present in the pomelo fruit membrane (PFM). These interactions can be developed by making hydrogen bonds. From this overall ferroelectric and dielectric properties of the PFM it can be expected that the piezoelectric performance of the PFM would be better. This independency of dielectric properties on the higher frequency level of the PFM indicate its self-polarization characteristics due to the higher number of dipoles. As a result, it shows better piezoelectric constant in the range of 3.2–4.6 pC/N with a frequency and dynamic force of 100 Hz and 0.05 N, respectively, measured by Piezometer (PM300) (images of piezometer are inserted in the supporting information Fig. S2).

### Energy harvesting performance of the PFM based nanogenerator

**Figure 4:**
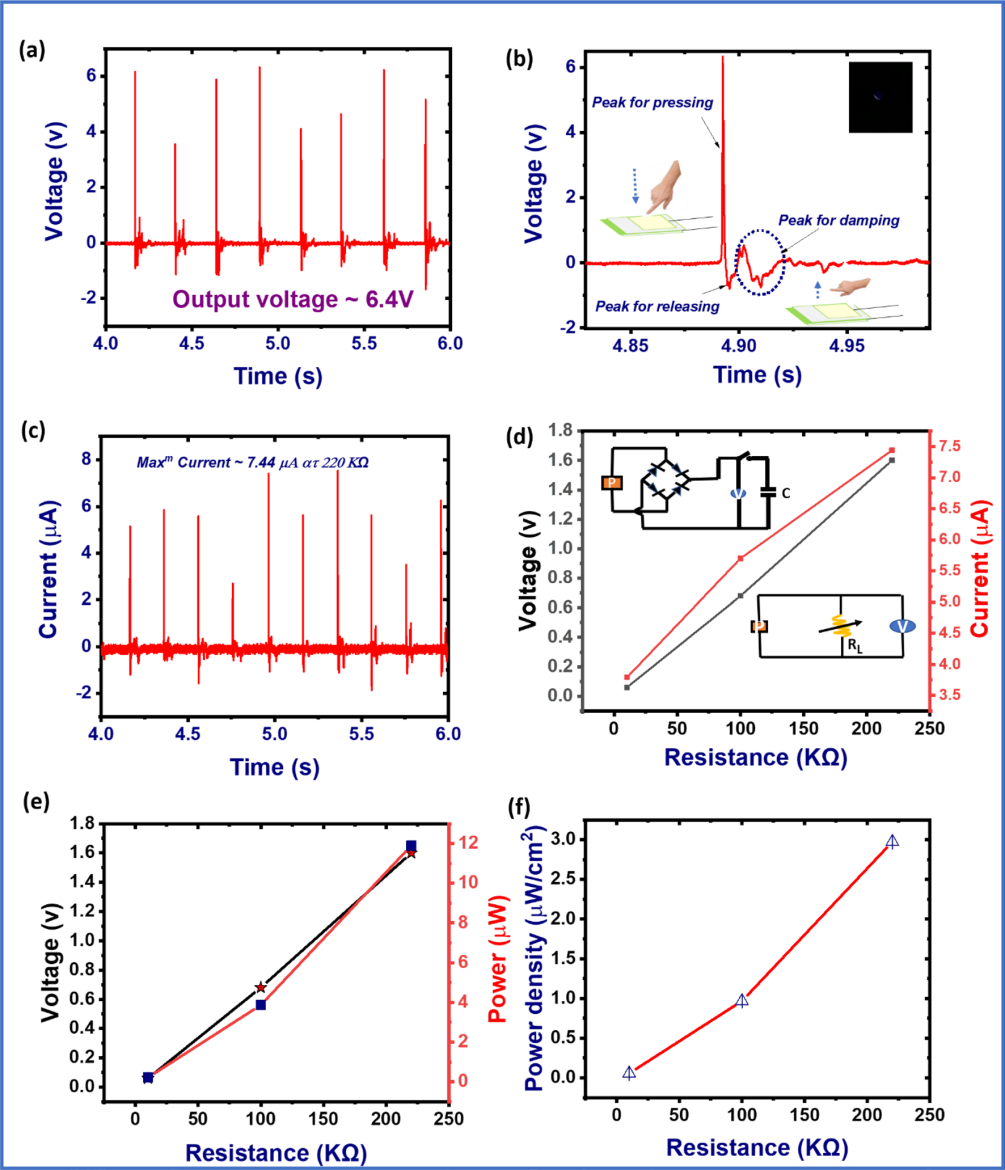
(**a**) Output voltage of the generator, (**b**) zoomed view of the selected part in (**a**), (**c**) output current of the generator at 220 kΩ resistance, (**d**) output voltage and current with different resistances, (**e**) voltage and power with different resistances, and (**f**) power density of the generator with respect to different resistances.

Figure 4a,b depict the output voltage generated from the pomelo fruit membrane bio-waste based piezoelectric generator (PFMBPEG) with simple finger tapping mode on the device. The magnitude of the applied pressure is ~ 1.1 kPa, as calculated by momentum and kinetic theorem^40^. It has been found that ~ 6.4 V output voltage has been generated by the PFMBPEG, as recorded by the digital oscilloscope. From the magnified view of the highlighted part of the Fig. 4a, the positive peak due to the applied pressure on the device and negative peak due to the release of the pressure from the device have been identified. Moreover, two weak output signals have also been observed in the magnified view (Fig. 4b) due to the elastic nature of the PFM^41^. This output voltage has been generated from the PFM, mainly due to the displacement and reorientation of the dipoles present in the crystal lattice of PFM by an application of pressure on the device^42^. Indeed, micro fibrils of cellulose, hemicellulose and pectin components of the pomelo fruit membrane act as a source of dipoles. These dipoles are basically formed by intra or inter molecular hydrogen bonds among the cellulose, hemicellulose and pectin components. The possible hydrogen bonds among these components are shown in the supporting information of this manuscript. As the number of hydroxyl, and carbonyl groups are higher in the case of PFM, the number of dipoles is higher. Therefore, piezoelectric response is also supposed to be higher as compared to the other reported bio-materials based piezoelectric generators.

The output current of the PFMBPEG has been calculated by using the output voltage generated across the 220 kΩ resistance connected in parallel with the generator as shown in Fig. 4c. The output current has been calculated by using the following formula:

3 $$V = IR$$

where, $V$ is the output voltage and $R$ is the resistance connected in series with the device, respectively.

**Figure 5:**
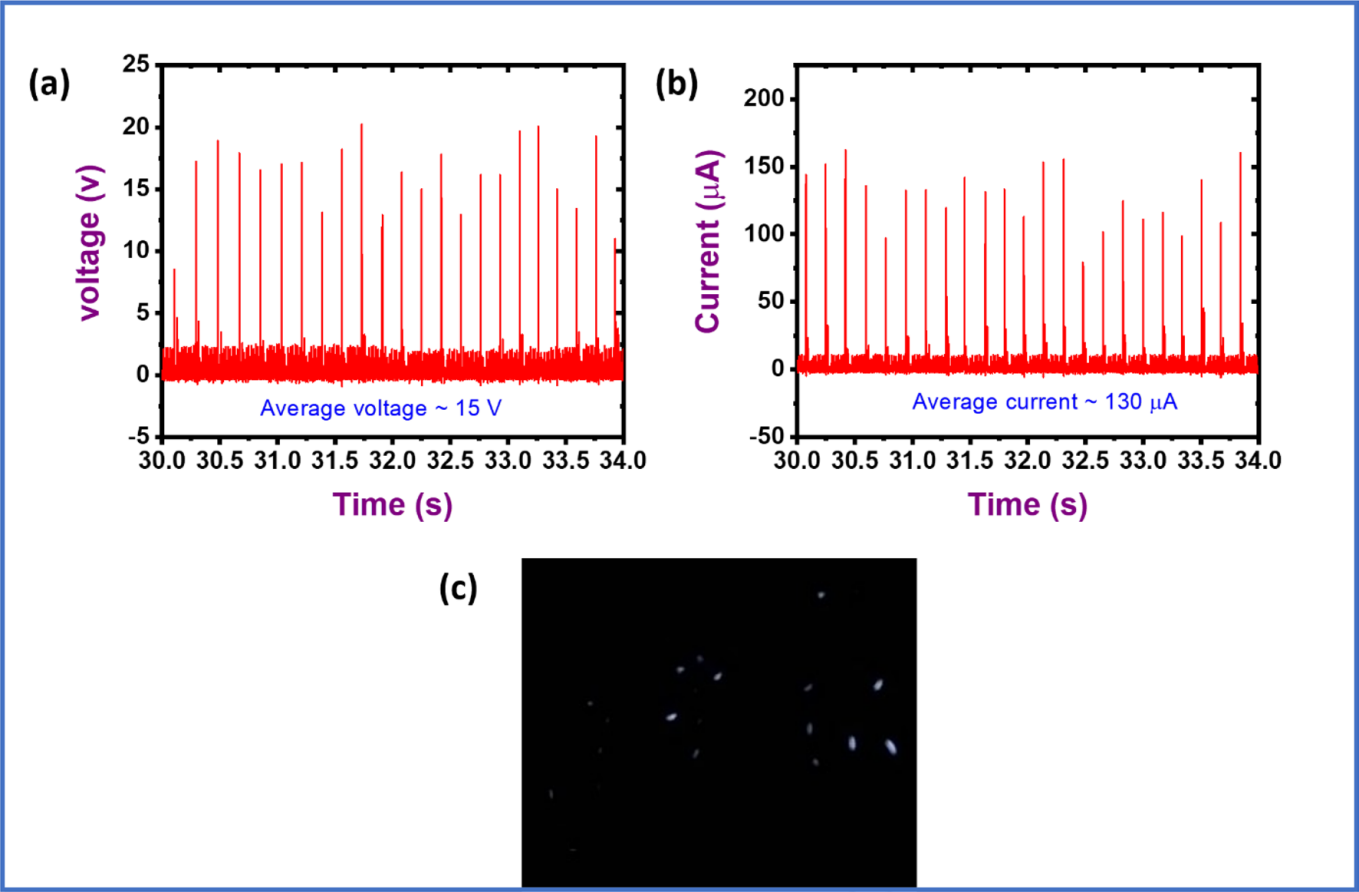
(**a**) Output voltage, (**b**) output current of the PFM based piezoelectric generator when a full wave rectifier was used to convert AC voltage to DC voltage, and (**c**) image of multiple LEDs lightened up by developed generator.

The output current of the PFMBPEG is ~ 7.44 μA with an external load resistance of 220 kΩ at 50 Hz excitation frequency. Figure 4d shows the output voltage and current of the PFMBPEG with respect to different capacity of resistance. It has been found that with increasing the resistance capacity, the output signal (voltage and current) of the device can be elevated. The respective circuit diagram for the current evaluation is shown in Fig. 4d (inset). The power (~ 48 μW) and power density (~ 12 μW cm^−2^) have also been calculated for the developed bio-waste based piezoelectric generator, at a resistance of 220 kΩ, respectively (calculation is given in supporting information). Figure 4e,f show the calculated power and power density with respect to different resistance levels (10 kΩ, 100 kΩ and 220 kΩ at 50 Hz excitation frequency). One can see from the figures that the power and power density have been increased by an increase in load. The piezoelectric properties of the developed PFMBPEG in terms of output voltage, current and power density have also been measured by using a full wave rectifier in the circuit. The full wave rectifier was used to convert AC voltage of the PFMBPEG into DC voltage. It can be seen from Fig. 5 that by using a full wave rectifier piezoelectric properties of the PFMBPEG device has been enhanced where the output voltage is ~ 15 V, output current is ~ 130 µA, and power density of ~ 487.5 μW cm^−2^. Also, to confirm the extent of power generation ability of the device, a multiple number of LEDs (2 V minimum voltage required to glow an LED) were made connected to this device. It was found that indeed the device is able to lighten a multiple number of LEDs as shown in Fig. 5c.

**Figure 6:**
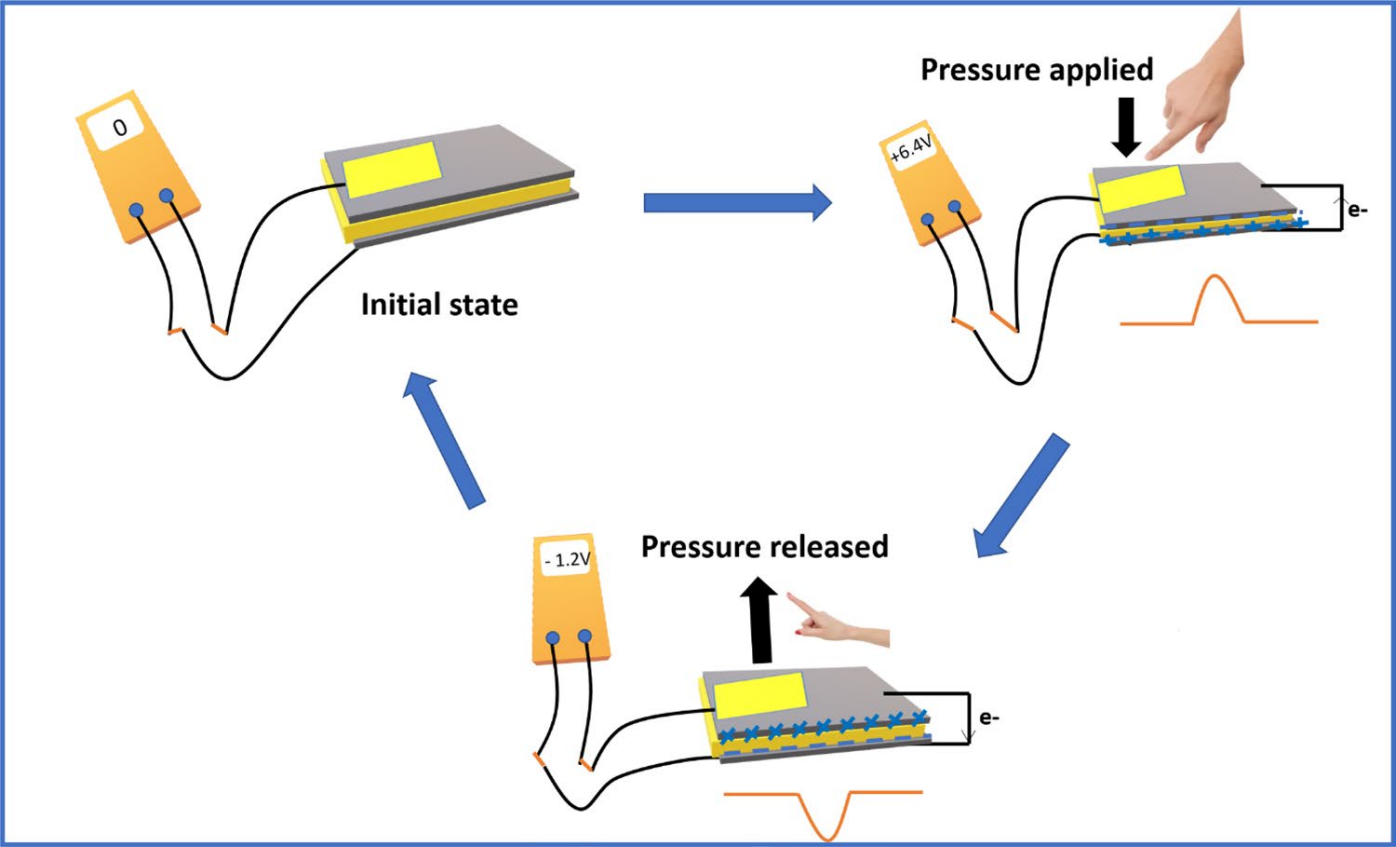
Schematic representation for working principle of PFMPEG under pressing and releasing the compression force.

The working principle of the PFMBPEG can be addressed in such a fashion that initially, the cellulose microfibrils, hemicellulose and pectin have been connected to each other or among themselves by forming a strong hydrogen bonds (the possible complex structure of the cellulose, hemicellulose and pectin have been depicted in supporting information). These hydrogen bonds have been responded as a dipole component in the PFM. When a pressure is applied on the device a stress or shear-induced polarization is built up in the vertical direction of the device. Therefore, all the positive and negative charges get accumulated in the top and bottom electrode and a potential difference creates between the electrodes. Because of this potential difference, charge carrier electrons flow from one electrode to another electrode via external resistor and as a result a big positive peak is observed. Again, when pressure is released from the device, all the stored charge carrier electrons flow to the opposite electrode for which a negative peak is observed as depicted in Fig. 6 ^43^. Therefore, with the repeated finger tapping on the device a sinusoidal output signal gets generated from the PFMBPEG, which can be confirmed from the output voltage signal as shown in Fig. 4a,b. PFMBPEG can be used to glow one light emitting diode (LED) as revealed in Fig. 4b at the inset.

### Evaluation of the energy harvesting feasibility and sensitivity of the generator

To further understand the feasibility to harness biomechanical energy and sensitivity of the developed PFMBPEG, different experimental trials have been performed. The detailed results and discussion have been discussed in below sections.

#### Energy harvesting from elbow, wrist, and fingers of the human body

**Figure 7:**
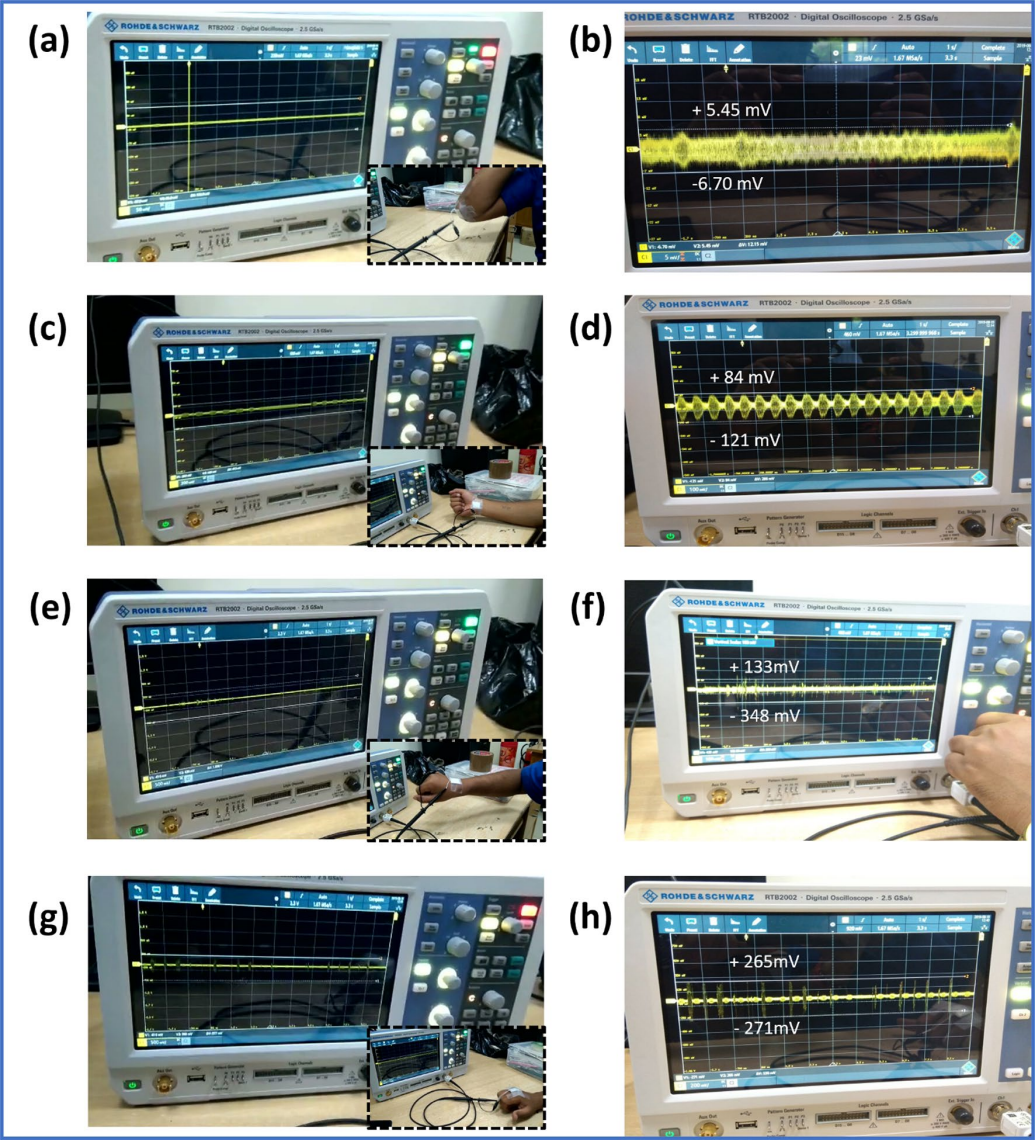
(**a**) Output voltage generation from the elbow motion (inset shows the device attached on the elbow), (**b**) enlarge view of the output signal depicted in (**a**), (**c**) output voltage generation from the front wrist motion (inset shows the device attached on the front wrist), (**d**) enlarge view of the output signal depicted in (**c**), (**e**) Output voltage generation from the back wrist motion (inset shows the device attached on the back wrist), (**f**) enlarge view of the output signal depicted in (**e**), (**g**) output voltage generation from the fingers motion (inset shows the device attached on the fingers), and (**h**) enlarge view of the output signal depicted in (**g**).

It is well known for us that a lump of mechanical energy has been wasted from the human body such as energy from the body movement, during walking, during speaking and so on. Therefore, scavenging of this waste bio-mechanical energy has become a challenge to the present researchers. Here, a small amount of energy has been generated from the developed PFMBPEG which comes from the elbow, wrist and finger movement of the human body. It has been found that this generator could generate ~ 12 mV (peak to peak) output voltage from the movement of elbow as depicted in Fig. 7a,b (inset shows the device attached on the elbow) (Supplementary video 4 has been included in Supporting information). When elbow is moving (bending and stretching), a tiny amount of stress is introduced in the device and it has been subjected to mechanical deformation. This mechanical deformation boosts to dipoles displacement and reorientation inside the PFM so that a potential difference takes place between the two electrodes. Due to this potential difference, electrons flow from one electrode to another electrode which provides output signal from the device as recorded by the digital oscilloscope. From this experiment it can be concluded that the developed bio-waste-based generator can successfully be utilized in powering small electronic gadgets or smart wearables. Furthermore, to investigate the energy harvesting feasibility of the PFMBPEG, one patch of the device has been attached on the wrist and finger, respectively. The generator patch has been attached on both sides (front and back) of the wrist and it has been found that the output signal of the generator shows a maximum value for the back wrist. This is due to the higher stress induced in the generator during movement of the wrist. The generator shows ~ 205 mV (Fig. 7c,d) and ~ 481 mV (Figs. 6f, 7e) output voltage from front wrist and back wrist movement, respectively (Supplementary videos 6 and 3 have been incorporated in supporting information).

To further reveal the energy harvesting ability of the generator, one device has been attached on the fingers and recorded the output voltage signal due to the bending and stretching of the fingers (Supplementary video 5 has been inserted in supporting information). One can see from the Fig. 7g,h that a peak to peak voltage of 536 mV has been generated by the PFMBPEG due to this movement. So, among the different realistic applications (elbow, wrist and fingers), the maximum output voltage has been generated from the finger. This can be due to the higher stress generation in the device by the movement (bending and stretching) of fingers.

From this above discussion, it can be manifested that the developed bio-waste based piezoelectric generators (PFEMBPEG) are able to harvest bio-mechanical energy very significantly.

#### Energy harvesting from the leg motion and ultrasonic bath vibration

**Figure 8:**
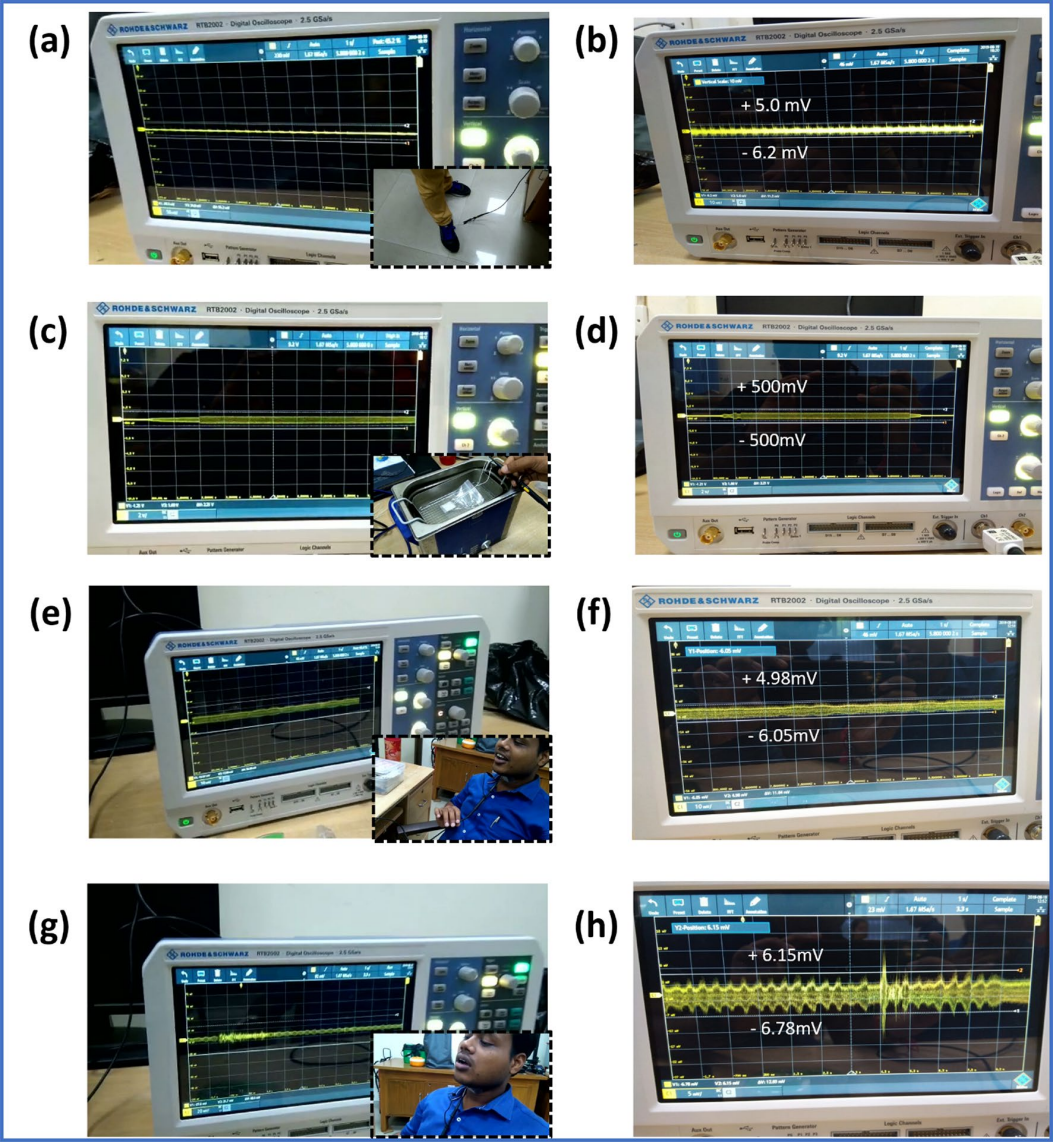
(**a**) Output voltage generation from the leg motion (inset shows the device attached below the shoe), (**b**) enlarge view of the output signal depicted in (**a**), (**c**) Output voltage generation from the ultrasonic vibration (inset shows the device placed in the ultrasonic bath), (**d**) enlarge view of the output signal depicted in (**c**), (**e**) output voltage generation from the vocal sound (inset shows the device attached on the throat), (**f**) enlarge view of the output signal depicted in (**e**), (**g**) output voltage generation from the jaws motion (inset shows the device attached on the throat), and (**h**) enlarge view of the output signal depicted in (**g**).

The energy harvesting performance of the developed bio-waste based piezoelectric generator (PFMBPEG) has also been evaluated by placing the device under the shoe and in the ultrasonic bath. Due to the movement of the leg, a pressure is applied on the generator and the same goes under minimal stress which results an output voltage of ~ 11 mV (peak to peak) as shown in Fig. 8a,b, (Supplementary video 7 has been attached as a supporting information). For further confirmation of the energy harvesting capability of the developed generator, an ultrasonic bath vibration has been used as a source of mechanical energy as depicted in Fig. 8c,d. When generator has been placed in the running ultrasonic bath a constant output voltage i.e., ~ 1 V has been observed from the recorded value of the digital oscilloscope. The output voltage can be seen to be maximum for the ultrasonic bath experiment than compared with the others. The reason for this might be due to the higher stress value which takes place in the device during the ultrasonic bath experiment. Indeed, the vibration in the ultrasonic bath spreads throughout the device for which maximum deformation take place into the device. As deformation is maximum, the displacement and reorientation of the dipoles inside the device also become maximum and the resultant dipole moment is also higher.

The sensitivity of the generator has also been evaluated by attaching the device on the throat. During speaking a vibration gets developed on the throat portion of a human being. Even this small vibration can be utilized as a usable energy by employing this nanogenerator. Here, a bio-waste based piezoelectric generator has been attached on the throat to evaluate the sensitivity of the generator. It has been found that with the continuous speaking condition this generator can generate a small output signal (~ 11 mV), result is shown in Fig. 8e,f (Supplementary video 8 has been added in supporting information). In addition, by jaws movement of the mouth, the sensitivity of the generator has also been tested. It can be seen from the Fig. 8g,h that an output voltage of ~  13 mV has been generated from the generator only from the jaw’s movement (Supplementary video 10 has been attached as a supporting information).

From this above-mentioned results and discussion, it can be concluded that the developed bio-waste based piezoelectric generator can easily be utilized as a bio-mechanical energy harvester. Moreover, for large scale application, a number of nanogenerators can be connected into series/parallel so that a higher amount of output voltage can be generated. This nanogenerator can also be used for the portable and smart wearable electronic goods where a small amount of energy is required to perform its activities.

### Analysis of mechanical stability of the piezoelectric device

**Figure 9:**
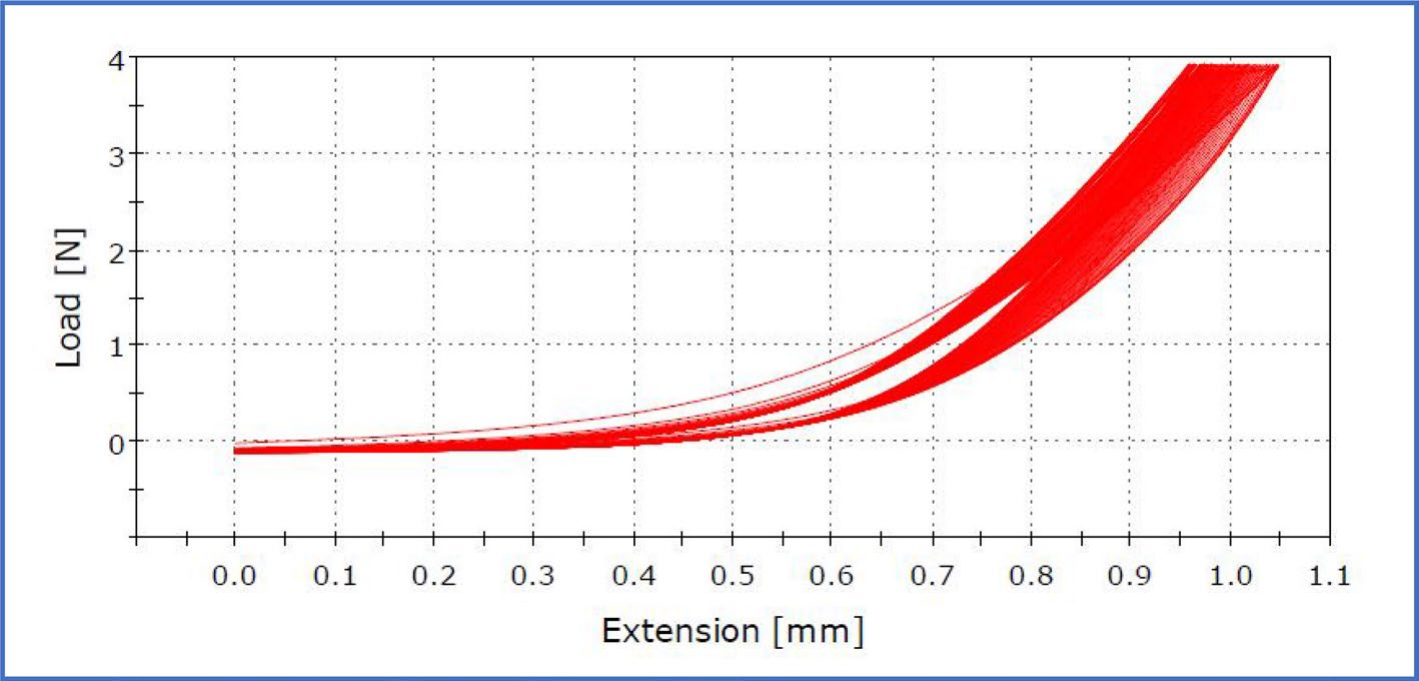
Cyclic tensile test of the PFM film at constant load of 400 gf.

Figure 9 shows the cyclic tensile test results of the pomelo fruit membrane (PFM). The testing was carried out using a universal tensile tester with a gauge length of 40 mm, testing speed of 1 mm min^−1^ and at a constant load of 400 gf for 100 cycles. From these results it can be seen that there was no significant deformation of the PFM film observed even after 100 cycles. Therefore, it can be concluded that this PFM based piezoelectric generator can be utilized for long time as per the application point of view. For instance, in this study, a force of 400 gf was applied on the PFM device for lightening the LEDs and thus similar force can also be used to power up other electronic gadgets as desired.

## Conclusion

In the present study, a green energy harvester based on Pomelo Fruit Membrane (PFM) has been fabricated very successfully which is sustainable, biocompatible, self-poled and consists of bio-waste-based material. The PFM has directly been used to fabricate the energy harvester without further treatment. The presence of different natural polymric chains such as cellulose, hemicellulose and pectin, are mainly responsible for the piezoelectric effect of this membrane. Peeled membrane from pomelo fruit was characterized to confirm its structure and revealed the source of piezoelectric properties. FTIR has been used to identify the functional groups (carboxylic, carbonyl and hydroxyl) present in the membrane, whereas XRD pattern has been used to calculate the total crystallinity of the membrane i.e., ~ 47%. The dielectric constant (ε) and remnant polarization (P_r_) values of the PFM have been noted as ~ 23.54 at 20 Hz frequency and ~ 0.27 μC cm^−2^, respectively. It has been observed that this energy harvester can directly generate ~ 6.4 V output voltage, ~ 7.44 μA current, and ~ 12 μW cm^−2^ power density, by finger tapping on the device. On the other hand, this device can generate ~ 15 V output voltage, ~ 130 μA output current, and ~ 487.5 μW cm^−2^ power density by connecting the device with a full wave rectifier. Finally, to investigate the practical usability and sensitivity of the generator, different green energy sources have been used. The generator has been attached at the elbow, wrist (front and back), fingers for harvesting the waste mechanical energy. The generator has also been placed below the shoe, on the throat and in the ultrasonic bath to test energy harvesting capability and sensitivity. It shows generation of energy in every case which is ~ 12 mV from the elbow bending, ~ 205 mV and ~ 481 mV from the front wrist and back wrist movement, respectively, ~ 536 mV from the fingers movement, ~ 11 mV from the leg motion, ~ 1 V from the ultrasonic bath wave, ~ 11 mV and ~ 13 mV from the vocal sound and jaws motion. To evaluate the mechanical stability of the developed PFM based device, a cyclic tensile test has also been performed. There was no significant deformation in the PFM film observed even after 100 cycles. Therefore, it can be summarized that PFM can be converted into a smart piezoelectric device as a sustainable power source which can be used for longer time multiple usage wherein various mechanical movements are the stimuli.

## Supplementary information

Supplementary video 1 (MP4 13549 kb)

Supplementary video 2 (MP4 7554 kb)

Supplementary video 3 (MP4 6016 kb)

Supplementary video 4 (MP4 5340 kb)

Supplementary video 5 (MP4 6637 kb)

Supplementary video 6 (MP4 5963 kb)

Supplementary video 7 (MP4 7583 kb)

Supplementary video 8 (MP4 4149 kb)

Supplementary video 9 (MP4 10155 kb)

Supplementary video 10 (MP4 4203 kb)

Supplementary information 1 (DOCX 1190 kb)

Supplementary video 11 (MP4 102077 kb)
