# Supplementary material for: A fully sustainable, self-poled, bio-waste based piezoelectric nanogenerator: electricity generation from pomelo fruit membrane
Source: Sci Rep. 2020 Jul 21;10:12121. doi: 10.1038/s41598-020-68751-3 (PMC7374593; doi:10.1038/s41598-020-68751-3)
Supplement: Supplementary file 11 — Supplementary information 1 (DOCX 1190 kb) [file 41598_2020_68751_MOESM11_ESM.docx]

**Supporting information**

**A Fully Sustainable, Self-Poled, Bio-waste based Piezoelectric Nanogenerator: Electricity Generation from Pomelo Fruit Membrane**

Satyaranjan Bairagi, Saikat Ghosh and S. Wazed Ali^#^

Department of Textile and Fibre Engineering

Indian Institute of Technology, Delhi, New Delhi-110016, India

*#Corresponding E-mail:* [*wazed@iitd.ac.in*](mailto:wazed@iitd.ac.in)*, Tel: +91-11-26597952; Fax: +91-11-26581103*

S1: Complex network among the cellulose, hemicellulose and pectin

The pomelo fruit membrane (PFM) is composed with a complex network structure of the cellulose, hemicellulose and pectin components, which is already mentioned in the main manuscript. Herein, this complex network is shown in Fig. S1. From the Fig.S1, one can see the intermolecular and intramolecular hydrogen bond formed which is denoted by dash line in the figure. Black dot line shows the bond formation within the cellulose chains whereas blue and red shows the bond between the cellulose and pectin and cellulose and hemicellulose, respectively.





Fig. S1 Schematic line diagram of the complex network in side the pomelo fruit membrane (PFM).

S2: d_33_ values for the pomelo fruit membrane (PFM)

The inherent dipoles in the PFM are helping to show the piezoelectric and ferroelectric properties. This dipole is coming from the strong hydrogen bonds formed between the -H atom of cellulose with -O atom of the same cellulose polymer or other side chain of the cellulose. The hydrogen bond can also be formed either between the cellulose and hemicellulose component or between the cellulose and pectin components. When PFM is placed in-between two copper electrode of the piezometer and start the instrument, a strain is applied in the 3 direction of the PFM for which a polarization is taking place in the same direction of PFM. Here, d_33_ values for the PFM are shown in Fig. S2.


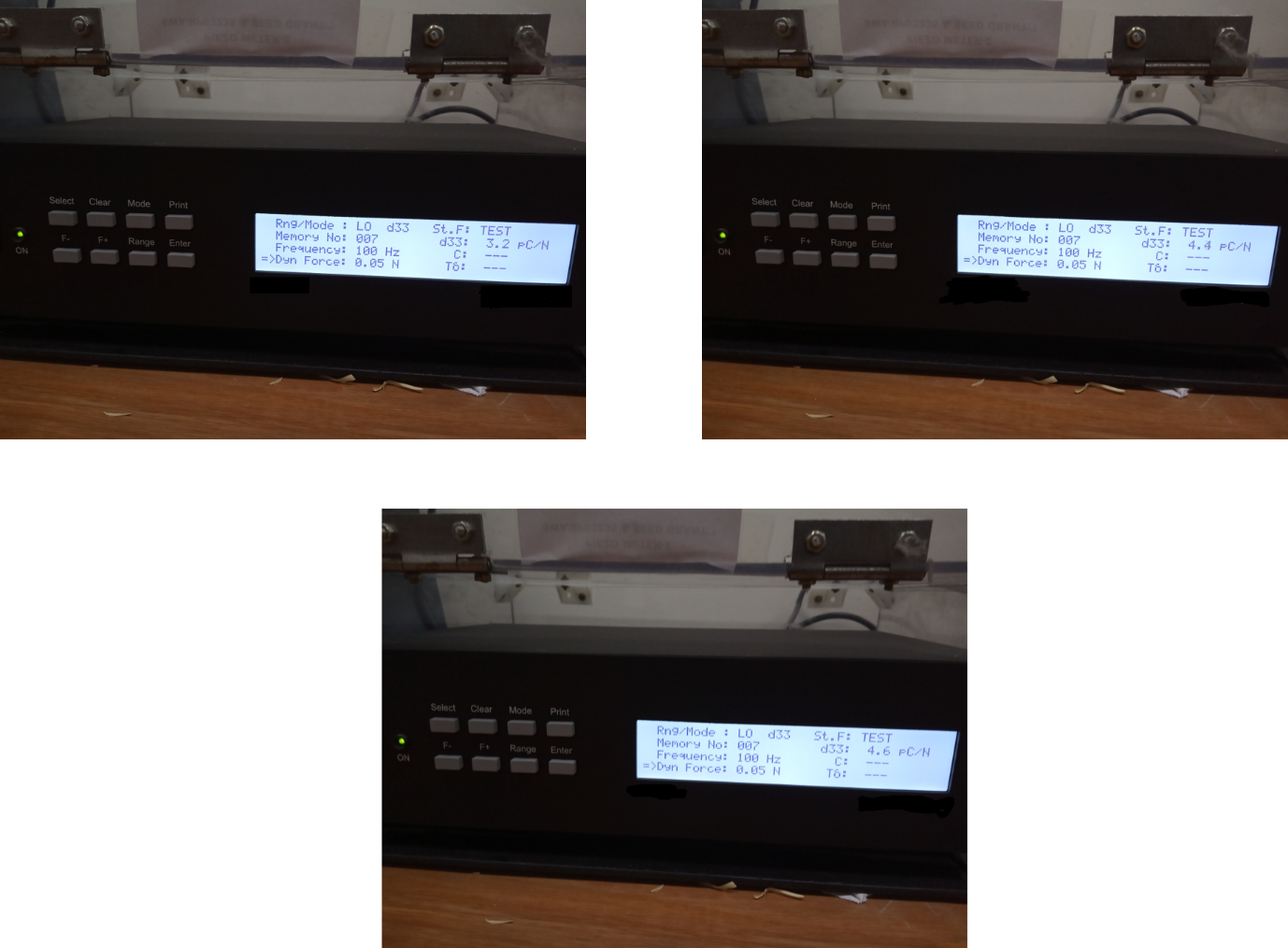


**Fig. S2** d_33_ value for the PFM
